# Cinacalcet versus standard treatment for chronic kidney disease: a protocol for a systematic review and meta-analysis

**DOI:** 10.1186/s13643-015-0177-1

**Published:** 2016-01-04

**Authors:** Nigar Sekercioglu, Jason W. Busse, Reem A. Mustafa, Gordon H. Guyatt, Lehana Thabane

**Affiliations:** Department of Clinical Epidemiology and Biostatistics, McMaster University, 1280 Main Street West, Hamilton, Ontario L8S 4K1 Canada; Department of Medicine, McMaster University, 1280 Main Street West, Hamilton, Ontario L8S 4K1 Canada; The Michael G. DeGroote Institute for Pain Research and Care, McMaster University, Hamilton, Canada; Department of Anesthesia, McMaster University, Hamilton, Canada; Departments of Internal Medicine and Biomedical & Health Informatics, University of Missouri–Kansas City, Kansas City, USA; Department of Pediatrics and Anesthesia, McMaster University, Hamilton, Canada; Centre for Evaluation of Medicine, St Joseph’s Healthcare Hamilton, Hamilton, Canada; Biostatistics Unit, Father Sean O’Sullivan Research Centre, St Joseph’s Healthcare, 3rd Floor, Martha Wing, 50 Charlton Avenue East, Hamilton, Ontario L8N 4A6 Canada; Population Health Research Institute, Hamilton Health Sciences, Hamilton, Canada

**Keywords:** Chronic kidney disease, Mineral and bone disorders, Secondary hyperparathyroidism, Calcimimetic agents

## Abstract

**Background:**

Chronic kidney disease-mineral and bone disorders (CKD-MBD) have been associated with poor health outcomes, including diminished quality and length of life. Standard management for CKD-MBD includes phosphate-restricted diet, active vitamin D, vitamin D analogs, and phosphate binders. Persistently elevated parathyroid hormone (PTH) levels may require the addition of Cinacalcet hydrochloride (cinacalcet) which sensitizes calcium receptors on the parathyroid glands. The objective of this systematic review is to compare the effect of cinacalcet versus standard treatment in patients with CKD-MBD.

**Methods/design:**

Data sources will include MEDLINE, EMBASE, the Cochrane Register of Controlled Trials, and Web of Science from 1996 to June 2015. Teams of two reviewers will, independently and in duplicate, screen titles and abstracts and potentially eligible full text reports to determine eligibility, and subsequently abstract data and assess risk of bias in eligible trials. We will calculate the effect estimates (risk ratios or mean differences) and 95 % confidence intervals, as well as statistical measures of variability in results across studies using random effect models for patient-important and intermediate outcomes. We will use the GRADE (Grading of Recommendations, Assessment, Development and Evaluation) approach to rate the quality of evidence about estimates of effect on an outcome-by-outcome basis. We will present our results with a GRADE summary table.

**Discussion:**

Our review will explore the effect of cinacalcet versus standard treatment in patients with CKD-MBD. The results of this systematic review will help guide management of this patient population, and identify targets for future research.

**Systematic review registration:**

PROSPERO CRD42015020318http://www.crd.york.ac.uk/PROSPERO/display_record.asp?ID=CRD42015020318.

## Background

Chronic kidney disease-mineral and bone disorder (CKD-MBD) is a systematic condition defined by three components: (1) extra skeletal calcifications; (2) abnormal metabolism of serum calcium, phosphorus, parathyroid hormone (PTH), and vitamin D; and (3) abnormal bone metabolism, including bone turnover, mineralization, linear growth, and reduced strength [1]. CKD-MBD is also associated with extravascular calcifications, increased cardiovascular events, and mortality [1]. The pathogenesis of CKD-MBD involves receptor level problems, including vitamin D receptors and calcium-sensing receptors [2, 3]. Abnormal serum calcium, phosphorus, PTH, and vitamin D levels also contribute to the development of the disease process [2–4].

Parathyroid gland hyperplasia is a common finding in patients with CKD-MBD, and proliferation in parathyroid cells leads to increase in parathyroid gland secretion. This leads to secondary hyperparathyroidism (SHPT) which is associated with elevated serum PTH levels [5]. A consensus exists regarding the need for CKD-MBD treatment to maintain guideline recommended targets for calcium, phosphorus, and PTH in the presumption that meeting these targets will improve quality and quantity of life [3, 6–8].

In the management of CKD-MBD, recommendations suggest that patients should, in addition to phosphate-restricted diet, receive active vitamin D and phosphate binders [3, 6, 9]. Medical management of persistent elevated PTH requires the use of calcimimetic agents. Cinacalcet hydrochloride (cinacalcet) is a second generation calcimimetic agent used to sensitize calcium receptors on the parathyroid glands [10, 11]. This leads to decreased PTH synthesis and secretion [10, 11]. Cinacalcet is a positive allosteric modulator that interacts with non-calcium binding sites [2]. Although this therapy has a very wide applicability, including parathyroid cancers and primary and secondary hyperparathyroidism, cost is a major drawback that limits utilization [12].

In 2004, the US Food and Drug Administration approved cinacalcet for uncontrolled SHPT in CKD-MBD patients. The medication is not recommended for predialysis patients because of the potential risks for severe hypocalcemia and phosphate increase due to the low PTH levels [6, 13, 14]. The total cost of cinacalcet per patient with CKD-MBD ranges from $4000 to $23,500 per year in Canada.

CKD-MBD has been associated with increased mortality, and reduced bone and cardiovascular health [15]. Prior systematic reviews have found that cinacalcet treatment reduces the rate of parathyroidectomy, fracture, and hospitalization due to cardiovascular events [16, 17]; the effect on mortality has not been established [18–20]. However, prior reviews have not included trials published after February 2013 and only included adult CKD patients.

We will update search covering more databases and the pediatric patient population with transparent quality assessment of findings. The consequences of CKD-MBD and pharmacokinetic and pharmacodynamic properties of cinacalcet may differ between the adult and pediatric patient populations. We will explore for a subgroup effect on this basis (adult versus children) and, if the test of interaction is significant, we will present results separately for these populations.

The objective of this systematic review is to evaluate the impact of cinacalcet treatment as compared to standard treatment alone or standard treatment plus placebo in patients with CKD-MBD and uncontrolled SHPT on patient-important outcomes, including cardiovascular events, fractures, parathyroidectomy, symptomatic hypotension, cardiovascular mortality, all-cause mortality, and intermediate outcomes—achieving Kidney Disease Outcome Quality Initiative targets (K/DOQI).

## Methods/design

### Data sources and search strategy

We will search the MEDLINE, EMBASE, and Cochrane Register of Controlled Trials and Web of Science databases from 1996 until June 2015 without any restrictions. We will use controlled vocabulary and text words to search all databases. We will scan the bibliographies of all prior systematic reviews and meta-analyses as well as all eligible primary studies for additional relevant articles. We will search for conference proceedings from 2010 to 2015 using the Web of Science and abstracts presented at recent annual meetings (the American Society of Nephrology, National Kidney Foundation, International Society of Nephrology and European Renal Association-European Dialysis and Transplant Association). Please refer to Table 1 for the full search strategy.

**Table 1:** Search strategy for the MEDLINE, EMBASE and Cochrane Central Register of Controlled Trials databases

	MEDLINE	
Set	History	Comments
1	(((kidney* or nephro* or renal or home or peritoneal or intermittent or chronic or extracorporeal or ambulatory) adj2 (haemodialys* or hemodialys* or dialys*)) or hemorenodialysis or hemodialyse or CAPD).ti,ab.	Dialysis textword search Terms
2	renal dialysis/ or hemodialysis, home/ or peritoneal dialysis/ or peritoneal dialysis, continuous ambulatory/	Dialysis subject Terms
3	renal insufficiency, chronic/ or kidney failure, chronic/	Chronic kidney disease subject terms
4	(((chronic or "end-stage" or "end stage") adj3 (kidney* or renal or nephro*) adj3 (insufficien* or disease*)) or esrd).ti,ab.	Chronic kidney disease textword terms
5	frasier syndrome/ or ("frasier* syndrome*" or (frasier* adj2 syndrome*)).mp.	Syndrome subject and textword terms
6	renalosteodystrophy/ or ((renal or kidney* or nephro*) adj2 (osteodystroph* or ricket*)).mp.	Chronic kidney disease subject or textword Terms
7	azotemia/ or azotemi*.mp.	Azotemia subject or textword Terms
8	uremia/ or uremi*.mp.	Uremia disease subject or textword Terms
9	or/1-1	Kidney disease terms
10	Calcimimetic Agents/ or (Calcimimetic* or amg073 or cinacalcet or "krn 1493" or krn1493 or mimpara or parareg or regpara or sensipar).mp.	Cinacalcet subject and textword search Terms
11	9 and 10	Base clinical set
12	controlled clinical trial.pt. or controlled clinical trials as topic/ or meta analysis.pt. ormeta analysis as topic/ or multicentre study.pt. or multicenter studies as topic/ or randomized controlled trial.pt. or randomized controlled trials as topic/ or pragmatic clinical trial.pt. or Pragmatic Clinical Trials as Topic/ or ((preference or practical or pragmatic or "real world" or naturalistic) adj5 trial*).ti,ab. or Comparative Effectiveness Research/ or ((comparative adj2 effectiveness) or (CER adj5 (research* or method* or framework* or compari* or statement*))).ti,ab. or ((singl: or doubl: or tripl: or trebl:) and (mask: or blind:)).ti,ab. or ((random: adj5 trial:) or rct or rcts).ti,ab.	Therapy Study design methodologies
13	11 and 12	FINAL Results
	EMBASE	
Set	History	Comments
1	(((kidney* or nephro* or renal or home or peritoneal or intermittent or chronic or extracorporeal or ambulatory) adj2 (haemodialys* or hemodialys* or dialys*)) or hemorenodialysis or hemodialyse or CAPD).ti,ab.	Dialysis textword search Terms
2	renal replacement therapy/ or continuous ambulatory peritoneal dialysis/ or continuous renal replacement therapy/ or extended daily dialysis/ or hemodialysis/ or home dialysis/ or peritoneal dialysis/	Dialysis subject Terms
3	kidney failure/ or chronic kidney failure/	Chronic kidney disease subject terms
4	(((chronic or "end-stage" or "end stage") adj3 (kidney* or renal or nephro*) adj3 (insufficien* or disease*)) or esrd).ti,ab.	Chronic kidney disease textword terms
5	frasier syndrome/ or ("frasier* syndrome*" or (frasier* adj2 syndrome*)).mp.	Syndrome subject and textword terms
6	renalosteodystrophy/ or ((renal or kidney* or nephro*) adj2 (osteodystroph* or ricket*)).mp. (7537)	Chronic kidney disease subject or textword Terms
7	azotemia/ or azotemi*.mp.	Azotemia subject or textword Terms
8	uremia/ or uremi*.mp.	Uremia disease subject or textword Terms
9	Or/1-8	Kidney disease terms
10	Calcimimetic Agent/ or cinacalcet/ or (Calcimimetic* or amg073 or cinacalcet or "krn 1493" or krn1493 or mimpara or parareg or regpara or sensipar).mp.	Cinacalcet subject and textword search Terms
11	9 and 10	Base clinical set
12	ct.fs. or controlled clinical trial.pt. or controlled clinical trial/ or meta analysis/ or multicenter study/ or randomized controlled trial.pt. or randomized controlled trial/ or double blind procedure/ or single-blind procedure/ or triple blind procedure/ or ((preference or practical or pragmatic or "real world" or naturalistic) adj5 trial*).ti,ab. or comparative effectiveness/ or ((comparative adj2 effectiveness) or (CER adj5 (research* or method* or framework* or compari* or statement*))).ti,ab. or ((random: adj5 trial:) or rct or rcts).ti,ab. or ((singl: or doubl: or tripl: or trebl:) and (mask: or blind:)).ti,ab.	Therapy Study design methodologies
13	11 and 12	FINAL Results
	EBM Reviews - Cochrane Central Register of Controlled Trials	
Set	History	Comments
1	((kidney* or nephro* or renal or home or peritoneal) adj2 (haemodialys* or hemodialys* or dialys*)).ti,ab. or renal dialysis/ or hemodialysis, home/ or peritoneal dialysis/ or peritoneal dialysis, continuous ambulatory/ or renal insufficiency, chronic/ or kidney failure, chronic/ or (((chronic or "end-stage" or "end stage") adj3 (kidney* or renal or nephro*) adj3 (insufficien* or disease*)) or esrd).ti,ab. or frasier syndrome/ or ("frasier* syndrome*" or (frasier* adj2 syndrome*)).mp. or renal osteodystrophy/ or ((renal or kidney* or nephro*) adj2 (osteodystroph* or ricket*)).mp. or azotemia/ or azotemi*.mp. or uremia/ or uremi*.mp. or (((kidney* or nephro* or renal or home or peritoneal or intermittent or chronic or extracorporeal or ambulatory) adj2 (haemodialys* or hemodialys* or dialys*)) or hemorenodialysis or hemodialyse or CAPD).ti,ab. or kidney failure/ or chronic kidney failure/ or frasier syndrome/ or renal osteodystrophy/ or uremia/ or (((chronic or "end-stage" or "end stage") adj3 (kidney* or renal or nephro*) adj3 (insufficien* or disease*)) or esrd).ti,ab. or kidney failure/ or chronic kidney failure/ or frasier syndrome/ or ("frasier* syndrome*" or (frasier* adj2 syndrome*)).mp. or renal osteodystrophy/ or ((renal or kidney* or nephro*) adj2 (osteodystroph* or ricket*)).mp. or azotemia/ or azotemi*.mp. or uremia/ or uremi*.mp.	Dialysis, chronic kidney diseases Subject or textword search Terms
2	Calcimimetic Agent/ or cinacalcet/ or Calcimimetic Agents/ or (Calcimimetic* or amg073 or cinacalcet or "krn 1493" or krn1493 or mimpara or parareg or regpara or sensipar).mp.	Cinacalcet subject and textword search Terms
3	1 and 2	Base clinical set

### Eligibility criteria

We will limit the studies included in this review to RCTs evaluating the effectiveness and safety of cinacalcet for the treatment of secondary HPT. The comparator will be the standard therapy alone or standard therapy plus placebo. The standard therapy includes phosphate binders and/or active vitamin D. This review will explore the effectiveness of cinacalcet treatment on the following outcomes: cardiovascular events, fractures, parathyroidectomy, symptomatic hypotension, cardiovascular mortality, all-cause mortality, end-of-treatment alkaline phosphatase, end-of-treatment PTH levels (any measure), end-of-treatment serum calcium concentrations (mg/dL or mmol/L), end-of-treatment serum phosphorus concentrations (mg/dL or mmol/L), end-of-treatment calcium x phosphorus product (mg^2^/dL^2^). We will not employ any restrictions for age. We will not include studies that the primary objective of which is to determine optimal dosing of cinacalcet and economic evaluation of cinacalcet treatment.

### Study selection

Teams of two investigators will independently screen each unique title and abstract that our literature search identifies. If either reviewer identifies a citation as potentially relevant, we will obtain the full text of the article. Two reviewers will independently determine the eligibility of all studies that undergo full text evaluation. We will measure the inter-rater agreement for full text eligibility and assessment of the risk of bias using the kappa statistic [21]. Values of kappa between 0.40 and 0.59 have been considered to reflect fair agreement, between 0.60 and 0.74 to reflect good agreement and ≥0.75 to reflect excellent agreement [21]. Disagreements will be resolved through discussion between the reviewers or through adjudication with a third party if necessary.

### Data abstraction

Using a standardized data collection form, two reviewers will collect the following information from each study: the author, date of publication, eligibility criteria, summary of baseline characteristics of the participants, number of participants in each arm at study onset and completion, duration of the trial, and treatment effects, including effectiveness and safety. We will resolve disagreements by discussion.

### Quality assessment of individual studies

A modified version of the Cochrane risk for bias tool (http://distillercer.com/resources/) will be employed by two independent reviewers in order to assess risk of bias on an outcome-by-outcome basis for all eligible trials. The assessment will include the following components: adequacy of sequence generation, allocation sequence concealment, level of blinding, incomplete outcome data, loss to follow-up, and stopping early for benefit or futility [22]. Reviewers will input response options of “definitely yes”, “probably yes”, “probably no”, and “definitely no” for each of the domains, with definitely yes and probably yes ultimately assigned low risk of bias and definitely no and probably no assigned high risk of bias [23]. Reviewers will resolve disagreements by discussion, and one arbitrator will adjudicate unresolved disagreements.

We will use to assess the quality of evidence for each outcome using the GRADE (Grading of Recommendations, Assessment, Development and Evaluation) rating system [24]. In the GRADE system of rating quality of evidence for each outcome, randomized trials begin as high quality evidence, but may be rated down by one or more of five categories of limitations [25]. We will use the GRADE methodology to rate certainty in effect estimates and quality of evidence for each outcome as high, moderate, low, or very low [25]. We will use the detailed GRADE guidance to assess the overall risk of bias, imprecision, inconsistency, indirectness, and publication bias and summarize results with a GRADE summary table [26]. The quality of evidence will be rated down, if we determine risk of bias as well as large imprecision, inconsistency, indirectness, and publication bias.

With the respect to precision, we will assess the optimal information size (OIS; the number of patients generated by a conventional sample size calculation for a single trial) and the width of the 95 % CIs. GRADE guidance notes that meta-analyses of small trials can provide evidence of benefit with 95 % CIs that appear to convincingly exclude no effect; however, the results of reviews of such studies have often been subsequently refuted by larger trials [27, 28]. To address this potential concern in cases in which our meta-analysis suggests benefit but the sample size is less than the OIS, we will rate down imprecision. For the purposes of calculating the OIS, we will assume, for binary variables a relative risk reduction or increase (delta) of 25 %, an alpha of 0.05, and a beta of 0.20, and a median baseline risk from the available studies. In addition, we will examine the width of 95 % CIs and verify if 95 % CIs overlap clinical decision thresholds for each outcome.

In addition to assessment of statistical heterogeneity, the degree of proximity in point estimates and overlap in 95 % confidence intervals (95% CI) will be examined to assess consistency between studies. Differences in methodology, study populations, outcomes, and quality and quantity of interventions will be considered to evaluate directness.

For each outcome, we will assess publication bias by visually observing asymmetry of the funnel plot for each outcome [29, 30]. We will follow published guidance and construct funnel plots only for outcomes with ≥10 trials. Publication bias will be considered as unlikely unless the effect measure is asymmetrically distributed around the pooled effect [29].

After considering these reasons for rating down, we will judge the quality of evidence for each outcome as follows: “high” quality of evidence (we are very confident that the true effect lies close to that of the estimate of the effect); “moderate” quality of evidence (we are moderately confident in the effect estimate and the true effect is likely to be close to the estimate of the effect, but there is a possibility that it is substantially different); “low” quality of evidence (our confidence in the effect estimate is limited and the true effect may be substantially different from the estimate of the effect); and “very low” quality of evidence (we have very little confidence in the effect estimate and the true effect is likely to be substantially different from the estimate of the effect) [25].

### Data synthesis and statistical analysis

We will report descriptive statistics as proportions for categorical variables and mean or medians for the continuous variables. We will calculate pooled risk ratios (RRs) and the associated 95 % CI for each outcome using random effects models by applying the Mantel-Haenszel method. Absolute effects and the associated 95 % CI will be calculated by multiplying pooled RRs and 95 % CI by the pooled control rate of outcomes. Statistical heterogeneity will be assessed by the chi-square test and *I*^2^ statistic. All data analyses will be performed using Stata (StataCorp. 2013. Stata Statistical Software: Release 13. College Station, TX: StataCorp LP).

### Dealing with missing participant data for dichotomous outcomes: sensitivity analyses

We will conduct a complete-case analysis for our primary analysis and perform sensitivity analyses to address the robustness of our findings with respect to the missing data. We plan to use plausible worst-case scenario for the missing trial-level data. The extent to which point estimates and 95 % CIs differ in these sensitivity analyses will determine whether we rate down for risk of bias [30, 31].

### Assessment of heterogeneity and subgroup analyses

Our estimates of study heterogeneity will be informed using the *p* value for the chi-square test for homogeneity and the *I*^2^ statistic where 0–40 % may be unimportant heterogeneity, 30–60 % moderate, 50–90 % substantial, and 75–100 % considerable heterogeneity [32]. If the *I*^2^ statistic indicates a value >50 %, we will explore heterogeneity. We will employ random effects meta-regression and include mean age, the mean baseline serum PTH concentration, trial duration, and stages of CKD (dialysis versus non-dialysis) in our univariate linear models.

We will employ subgroup analysis for the pediatric and adult patient populations. If the interaction test suggests a significant subgroup effect, we will report effect estimates for each subgroup separately.

### Presentation of results

We will present the results of our meta-analyses in an evidence profile that will provide a succinct, easily digestible presentation of quality of evidence, and magnitude of effects [26]. Our evidence profile will be constructed to include the following elements: a measure of the typical burden of these outcomes (e.g., control group, estimated risk; if appropriate studies are available, we will use the baseline risk for population-based observational studies); a measure of the absolute risk difference between the risks with and without intervention; the relative magnitude of effect; the numbers of participants and studies addressing these outcomes; a GRADE rating of the overall confidence in estimate of effect for each outcome and any reasons for rating down the confidence [32].

## Discussion

We expect to provide an objective assessment of effectiveness and safety of cinacalcet in patients with CKD-MBD. We will examine the impact of cinacalcet on patient-important outcomes. In addition, we will explore the quality of the existing evidence in both adult and pediatric patient populations with CKD-MBD. This will be the first systematic review that includes both pediatric and adult CKD patient populations in the assessment of cinacalcet and patient important outcomes available in the literature. We will disseminate our results in local meetings and in a peer-reviewed publication.

### Strengths and limitations of this study

The methods of our proposed review are state of the art, including explicit eligibility criteria, a comprehensive search, independent duplicate assessment of eligibility, and the use of the GRADE approach to assessing quality of evidence of effect including independent duplicate assessment of risk of bias, precision, consistency, directness, and publication bias. Our protocol represents a model for systematic review methods. Our results are likely to be limited by limitations in the primary studies.

## Abbreviations

Ca: calcium
CKD-MBD: chronic kidney disease-mineral and bone disorders
GRADE: Grading of Recommendations, Assessment, Development and Evaluation
OIS: optimal information size
P: phosphate
PTH: parathyroid hormone
RCT: randomized controlled trail
RR: risk ratios
SHPT: secondary hyperparathyroidism
